# Dual probe difference specimen imaging for prostate cancer margin assessment

**DOI:** 10.1117/1.JBO.28.8.082806

**Published:** 2023-04-18

**Authors:** Marcus J. Kwon, Broderick J. House, Connor W. Barth, Allison Solanki, Jocelyn A. Jones, Scott C. Davis, Summer L. Gibbs

**Affiliations:** a Oregon Health & Science University, Biomedical Engineering Department, Portland, Oregon, United States; b Knight Cancer Institute, Oregon Health & Science University, Portland, Oregon, United States; c Thayer School of Engineering at Dartmouth College, Hanover, New Hampshire, United States

**Keywords:** dual probe difference specimen imaging, prostate cancer, prostatectomy, tumor margin assessment, fluorescence guided surgery

## Abstract

**Significance:**

Positive margin status due to incomplete removal of tumor tissue during radical prostatectomy for high-risk localized prostate cancer requires reoperation or adjuvant therapy, which increases morbidity and mortality. Adverse effects of prostate cancer treatments commonly include erectile dysfunction, urinary incontinence, and bowel dysfunction, making successful initial curative prostatectomy imperative.

**Aim:**

Current intraoperative tumor margin assessment is largely limited to frozen section analysis, which is a lengthy, labor-intensive process that is obtrusive to the clinical workflow within the operating room (OR). Therefore, a rapid method for prostate cancer margin assessment in the OR could improve outcomes for patients.

**Approach:**

Dual probe difference specimen imaging (DDSI), which uses paired antibody-based probes that are labeled with spectrally distinct fluorophores, was shown herein for prostate cancer margin assessment. The paired antibody-based probes consisted of a targeted probe to prostate-specific membrane antigen (PSMA) and an untargeted probe, which were used as a cocktail to stain resected murine tissue specimens including prostate tumor, adipose, muscle, and normal prostate. Ratiometric images (i.e., DDSI) of the difference between targeted and untargeted probe uptake were calculated and evaluated for accuracy using receiver operator characteristic curve analysis with area under the curve values used to evaluate the utility of the DDSI method to detect PSMA positive prostate cancer.

**Results:**

Targeted and untargeted probe uptake was similar between the high and low PSMA expressing tumor due to nonspecific probe uptake after topical administration. The ratiometric DDSI approach showed substantial contrast difference between the PSMA positive tumors and their respective normal tissues (prostate, adipose, muscle). Furthermore, DDSI showed substantial contrast difference between the high PSMA expressing tumors and the minimally PSMA expressing tumors due to the ratiometric correction for the nonspecific uptake patterns in resected tissues.

**Conclusions:**

Previous work has shown that ratiometic imaging has strong predictive value for breast cancer margin status using topical administration. Translation of the ratiometric DDSI methodology herein from breast to prostate cancers demonstrates it as a robust, ratiometric technique that provides a molecularly specific imaging modality for intraoperative margin detection. Using the validated DDSI protocol on resected prostate cancers permitted rapid and accurate assessment of PSMA status as a surrogate for prostate cancer margin status. Future studies will further evaluate the utility of this technology to quantitatively characterize prostate margin status using PSMA as a biomarker.

## 1 Introduction

Prostate cancer is consistently among the most diagnosed cancers in men, with >250,000 new cases expected in the United States in 2022.^1^ Currently, the primary definitive therapy for localized prostate cancer is radical prostatectomy,^2^ which can be curative if all cancer is removed. Thus, the success of prostatectomy is reliant upon negative cancer margins, which is essential to decrease patient morbidity and mortality. Prostatectomies with positive margins, as determined by histopathology, necessitate further adjuvant therapies including combination treatments of radiation, androgen deprivation therapy (ADT), chemotherapy, and/or bone-directed therapy (e.g., zoledronic acid or denosumab).^3^ Long-term follow-up studies have shown that >95% of patients with prostate cancer who received prostatectomy or radiation had erectile dysfunction, and ∼50% reported urinary incontinence or bowel dysfunction, further emphasizing the importance of improved surgical treatment.^4^ Current intraoperative margin detection protocols are largely limited to rapid frozen section analysis (FSA), which has not received widespread clinical adoption due to the necessity for a pathologist within the operating room (OR), a staining protocol that increases surgical time, and significant variation in sensitivity and specificity between studies.^5^ Due to these challenges, intraoperative FSA has not been widely adopted in U.S. hospitals for margin assessment during prostatectomy, leaving an unmet clinical need for a methodology that is able to rapidly improve margin detection within the OR.

A variety of technologies are under development to improve intraoperative margin assessment including Raman spectroscopy using endogenous tissue contrast as well as microscopy with ultraviolet (UV) surface excitation to enable rapid slide-free histology, further highlighting this unmet clinical need.^6^^,^^7^ With the continuing advancement of fluorescent guided surgery (FGS), an optical imaging modality with real-time imaging capabilities, there is already great promise for FGS to become integrated into the intraoperative workflow.^8^^–^^10^ Fluorescent molecular tracers to guide cancer resections for a variety of cancer types including prostate, bladder, colorectal, pancreatic, brain, head and neck, breast, skin, lung, cervical, esophageal, and renal cancers are under development and clinical translation.^11^^–^^33^ Furthermore, the first molecularly targeted fluorophore, pafolacianine, has recently received Food and Drug Administration approval for use in ovarian and lung cancer, further cementing FGS as part of the armamentarium of tools to improve cancer care. Although *in vivo* administration of fluorescent contrast agents to patients is appealing since it enables real-time imaging guidance within the surgical field of view, there are still limited clinically approved contrast agents since the required approval process for new agents is both lengthy and costly.^34^^,^^35^ Topical application of tumor-specific fluorescent probes on the resected specimen offers a conceptually simpler approach without the challenges of *in vivo* administration. Particularly, in the case of surgical cancer removal, where the goal is margin negative resection, staining of the resected specimen is an attractive alternative to enable molecular-specific margin assessment without the need for the contrast agent to touch the patient while leveraging the existing FGS clinical infrastructure.

Although conceptually simple, topical targeted probe administration to resected tissues is dominated by nonspecific uptake and thus fluorescent signal in both the tumor tissue and surrounding normal tissues, negating the bulk of biomarker targeted contrast. Notably, use of an untargeted, companion probe of equivalent size and charge can effectively map this nonspecific tissue uptake, which shows substantial intra- and intertissue variability. The use of targeted and untargeted companion probe staining has previously been investigated using antibodies labeled with spectrally distinct fluorophores and surface enhance Raman scattering (SERS) nanoparticles.^36^^–^^41^ To facilitate quantitative biomarker-specific imaging, the targeted and untargeted probes have spectrally distinct labels (i.e., fluorophores or SERS nanoparticles), enabling the targeted and untargeted probe distribution to be quantified using multiplexed detection. The normalized difference between the targeted and untargeted probes’ signals emphasizes the difference between each probe’s uptake and enhances the signal difference between the tumor that overexpresses the targeted biomarker and the normal tissue with minimal biomarker expression. The resulting calculated image is then a quantitative representation of the targeted probes binding to the specific biomarker. We have termed this technique dual probe difference specimen imaging (DDSI), which has been optimized using spectrally distinct, fluorescently labeled targeted and untargeted antibodies.

A robust DDSI protocol with high sensitivity and specificity for differentiation between benign and malignant breast tissues^36^^,^^37^^,^^42^^–^^44^ was previously developed and optimized using antibody-based probes on resected specimens to a total staining and imaging time of 8 min for routine intraoperative use with minimal disruption to the clinical workflow. With advancements in FGS technologies and increasing clinical adoption, the overall goal of this study was to expand the DDSI tumor margin assessment methodology to other cancer types, where prostate cancer was evaluated herein. Given the development in nuclear medicine and recent fluorescent agents for prostate cancer using prostate-specific membrane antigen (PSMA),^45^^,^^46^ it was selected as the prostate cancer-specific target in this study. PSMA is a glycosylated type II transmembrane protein that is overexpressed in >90% of all primary prostate cancer lesions, tumor-positive lymph nodes, and metastases^47^^–^^51^ and is the most well established, highly specific prostate epithelial cell membrane antigen.^52^^–^^54^ In addition, its expression is positively correlated with prostate cancer grade (i.e., Gleason grade), where higher-grade cancers have higher levels of expression, and expression is minimal in healthy tissues.^55^^,^^56^ Herein, PSMA was used to examine the diagnostic accuracy of the DDSI method in prostate cancer, where receiver operator characteristic (ROC) curve analysis was used to quantify diagnostic performance. Ultimately, adoption of a clinically relevant DDSI protocol could enable rapid intraoperative cancer margin assessment in prostate cancer at the time of surgery without administration of the fluorescently labeled probes to the patient while preserving tissue integrity for gold standard histopathology enabling rapid clinical translation.

## 2 Methods

### 2.1 Fluorophores & Antibodies

Alexa Fluor 647 (AF647; Thermo Fisher Scientific, Waltham, Massachusetts, United States) and Cy3B (GE Healthcare Life Sciences, Little Chalfont, United Kingdom) were purchased in their N-hydroxysuccinimide ester form and then solubilized in anhydrous dimethyl sulfoxide (DMSO) at 10 mM for antibody conjugation. For the targeted probe, PSMA targeted monoclonal antibody (Mab) J533 IgG was used. The mouse hybridoma Prost J533 (HB-12127) was purchased from American Type Culture Collection (ATCC; Manassas, Virginia, United States). The hybridoma was used to produce the J533 antibody as the IgG, K isotype, which was purified using mercaptoethylpyridine/protein A chromatography by the Monoclonal Antibody Core at Oregon Health and Science University (OHSU). For the untargeted probe, donkey anti-rabbit (DkRb) IgG (Jackson ImmunoResearch, West Grove, Pennsylvania, United States) was used.

### 2.2 Cell Lines

The human prostate carcinoma cell line LNCaP clone FGC was cultured in RPMI 1x (Gibco/Thermo Fisher Scientific, Waltham, Massachusetts, United States) with 10% fetal bovine serum ([FBS], Seradigm, Sanborn, New York, United States) and 1% penicillin–streptomycin–glutamine (Thermo Fisher Scientific). The human prostate grade IV adenocarcinoma cell line PC-3 was cultured in F-12K 1x (Gibco/Thermo Fisher Scientific) with 10% FBS and 1% penicillin–streptomycin–glutamine. All cell lines were cultured to ∼90% confluence prior to use.

### 2.3 Antibody-Fluorophore Conjugation

The targeted probe J533 IgG was conjugated to AF647, and the untargeted probe DkRb IgG was conjugated to Cy3B. Each probe was prepared individually, explained briefly as follows. First, the antibodies were buffer exchanged into 1x phosphate-buffered saline (PBS) at pH 8.0. Following this, 10 mM AF647 in anhydrous DMSO was added to the J533 IgG, and 10 mM Cy3B in anhydrous DMSO was added to the DkRb IgG, resulting in a 5:1 fluorophore-to-antibody molar ratio in a total volume of 1 mL for each solution diluted in 1x PBS at pH 8.0. The solutions were shaken gently for 3 h at room temperature (RT), protected from light. The resulting solutions were then concentrated in 10 kDa molecular weight cut-off spin filters (Amicon Ultra 0.5 mL 10 kDa, Fisher Scientific) into clean microcentrifuge tubes to remove any unconjugated fluorophore. The fluorophore-to-protein ratio for each conjugated antibody was quantified using absorbance spectroscopy (SpectraMax M5 Microplate Reader, Molecular Devices, Sunnyvale, California, United States). Antibody absorbance was measured at 280 nm (J533 and DkRb IgG estimated extinction coefficient $(\varepsilon )=210,000\;M^{-1}\;{\mathrm{cm}}^{-1}$). AF647 absorbance was measured at 650 nm (AF647 $\varepsilon =270,000\;M^{-1}\;{\mathrm{cm}}^{-1}$). Cy3B absorbance was measured at 560 nm (Cy3B $\varepsilon =130,000\;M^{-1}\;{\mathrm{cm}}^{-1}$). Calibrated absorbance and fluorescence spectra and the Beer–Lambert law were used to determine the final concentrations of fluorophore and antibody for each conjugated antibody. All antibody conjugates had a fluorophore-to-antibody ratio of ∼2∶1.

### 2.4 Flow Cytometry

LNCaP and PC-3 cells were trypsinized, counted, and resuspended to $1\times 10^{7}$ cells each in 1 mL of 1x PBS. Cells were then fixed in 4% paraformaldehyde (PFA) for 10 min, followed by 3-min permeabilization in 1x PBS + 0.1% Tween-20. The cells were then washed with 1x PBS and blocked with 5% FBS in 1x PBS for 15 min. 20  μg/mL of the J533-AF647 conjugate was added to each sample resulting in a final staining concentration of 10  μg/mL. The cells were washed with 1x PBS +0.5% Triton-X for 5 min, followed by two washes with 1x PBS for 5 min each. The cells were resuspended in 500  μL of fresh 1x PBS immediately prior to analysis on a Becton Dickinson LSR Fortessa (Becton, Dickinson and Company, Franklin Lakes, New Jersey, United States). To detect AF647, the flow cytometer was configured to the 640-1 (650/665) Cy5 channel. A minimum of $1\times 10^{5}$ cells were counted for each sample. To quantify PSMA receptor number, Quantum^TM^ AF647® molecules of equivalent soluble fluorophore beads (Bangs Laboratory, Inc., Fishers, Indiana, United States) were quantified prior to the LNCaP and PC-3 PSMA stained samples.

### 2.5 Spinning Disk in Vitro Immunofluorescence

LNCaP and PC-3 cells were trypsinized, counted, and resuspended to a final concentration of $1.5\times 10^{5}$ cells per well into a 96 well glass bottom plate (Cellvis P96-1.5H-N, Mountain View, California, United States). Cells were fixed with 4% PFA for 15 min at RT, followed by two washes with 1x PBS for 5 min each at RT. Cells were then blocked with 5% FBS in 1x PBS for 15 min at RT. Blocking solution was then carefully removed without additional washing. The cells were then stained with the J533-AF647 conjugate for 30 min at RT with final staining concentrations of 1, 5, 10, and 20  μg/mL. Cells were washed with 1x PBS + 0.5% Triton-X for 5 min at RT, followed by a wash with 1x PBS for 5 min at RT. Cell staining with the J533-AF647 conjugate was compared to an unstained control sample. A Hoechst nuclear stain (Thermo Fisher Scientific) was performed on all samples with a final staining concentration of 1  μg/mL. The cells were then imaged at 20× on a Yokogawa CSU-X1 Zeiss Axio Observer microscope (Oberkochen, Germany) in the UV and Cy5 channels.

### 2.6 Mice

Separate cohorts of male athymic nude mice (32 to 38 days old, Homozygous 490, Charles River Laboratories, Wilmington, Massachusetts, United States) weighing 19 to 21 g were used to grow LNCaP and PC-3 tumor xenografts. All animal studies were approved by the Institutional Animal Care and Use Committee at OHSU (Protocol No. TR01_IP0000202).

### 2.7 Tumor Implantation and Growth

All mice were anesthetized with a 100 mg/kg ketamine (Hospira Inc., Lake Forest, Illinois, United States) and 10 mg/kg xylazine (AnaSed, Shenandoah, Indiana, United States) solution injected intraperitoneally. The toe pinch method was utilized to ensure the mice were fully anesthetized prior to tumor implantation. The lower peritoneal region of each mouse was prepared in a sterile field using povidine-iodine (Purdue Products, Stamford, Connecticut, United States). For LNCaP and PC-3 xenografts, 200  μL of cell suspension in fresh media ($1\times 10^{6}\;\mathrm{cells}$) was injected into both hind flanks of each mouse subcutaneously. The mice were monitored weekly following implantation for tumor growth and general well-being.

### 2.8 Tumor Resection & DDSI Staining

All mice were euthanized via carbon dioxide asphyxiation followed by cervical dislocation prior to tumor resection, which is compliant with the recommendations by the panel on euthanasia of the American Veterinary Medical Association. LNCaP tumor bearing mice were euthanized after 6 weeks of tumor growth or a maximum tumor size of $1\;{\mathrm{cm}}^{3}$. PC-3 tumor bearing mice were euthanized after 4 weeks of tumor growth or a maximum tumor size of $1\;{\mathrm{cm}}^{3}$. For each cell line, two cohorts of n=6 tumors were grown in n=3 mice and were extracted for DDSI staining. Thus, in total, n=12 tumors per tumor type were grown in n=6 mice per tumor type. Each extracted tumor was bisected for DDSI staining, where the cut faces were imaged. For the first cohort of mice, corresponding normal tissues, including a peritoneal adipose sample and a peritoneal muscle sample, were extracted from each mouse for comparison. For the second cohort of mice, corresponding normal tissues, including a peritoneal adipose sample, a peritoneal muscle sample, and normal prostate tissue, were extracted from each mouse for comparison. Each bisected LNCaP and PC-3 tumor sample was blocked, stained, and washed together in a single Eppendorf tube according to an optimized, previously published procedure,^37^ explained briefly as follows. Each xenograft type was blocked with 1 mL of 5% bovine serum albumin (BSA) solution in 1x PBS at pH 7.4 for 2 min, stained with 1 mL of 200 nM J533-AF647 + 200 nM DkRb-Cy3B solution in 1x PBS at pH 7.4 containing 1% BSA and 0.1% Tween-20 for 1 min, and then washed in 50 mL of 1x PBS containing 0.1% Tween-20 for 3 min. The tissue samples were imaged immediately after conclusion of the wash step with the bisected tumor cut-face oriented towards the light source and camera.

### 2.9 DDSI Macroscopic Imaging

White light and fluorescence images of tumor, adipose, muscle, and prostate tissues were collected using a previously described custom-built wide-field fluorescence imaging system consisting of a QImaging EXi Blue monochrome camera (Surrey, British Columbia, Canada) for fluorescence detection with a removable Bayer filter and a near infrared (NIR) PhotoFluor II light source (89 North, Burlington, Vermont, United States).^57^ The broad band light source (360 to 800 nm) was filtered using a 545±12.5  nm or a 620±30  nm bandpass excitation filter for fluorescence excitation of Cy3B and AF647, respectively. Fluorescence images were collected with a 605  nm±35  nm or a 700  nm±37.5  nm bandpass emission filter for Cy3B or AF647, respectively. All filters were obtained from Chroma Technology (Bellows Falls, Vermont, United States). To facilitate image calibration, an aliquot of the staining solution was placed in a covered optical well plate (Greiner Bio-One, Monroe, North Carolina, United States) and imaged at the beginning of each DDSI tissue staining experiment.

### 2.10 DDSI Image Processing

The collected targeted and untargeted fluorescence images were processed using custom written MATLAB code (MathWorks, Natick, Massachusetts, United States) to generate DDSI maps of each set of tumor, adipose, muscle, and prostate tissues described as follows. First, the median background signal from a user-selected region of interest (ROI) absent of resected tissue was subtracted from the entire image. Separate ROIs were then selected within an equal volume of each probe in solution that was imaged in a 96 well plate to calculate the mean intensity value of the targeted and untargeted staining solutions. These values were subsequently used to normalize the targeted and untargeted images permitting calculation of DDSI tissue map. Each pixel value within the ROI in both targeted and untargeted fluorescence images was divided by the average intensity value of the ROI representative of the DDSI staining solution in their respective imaging channels. Each tissue sample ROI was selected to encompass the bulk of tissue without containing any of the tumor edges so as to ensure that all pixels selected corresponded to the tissue type. User-created manual masks were used to identify tissue sample ROIs containing tumor or normal (i.e., adipose, muscle, or prostate) tissues based on white light images alone to minimize selection bias that could come from processing the fluorescence images. These user-created manual masks on white light images were overlaid onto each normalized fluorescence image (targeted and untargeted), and final DDSI images were then calculated as $I_{\text{DDSI}}=(I_{\text{Targeted}}-I_{\text{Untargeted}})/I_{\text{Untargeted}}$, where I = signal intensity/pixel.

### 2.11 H&E Staining and Immunohistochemistry

Following DDSI, each tumor and normal tissue set were flash frozen in optical cutting temperature compound to preserve the tissue and its orientation. Frozen blocks were sectioned at 10  μm thickness onto superfrost plus slides, where serial sections were used for immunofluorescence (IF) and gold standard hematoxylin and eosin (H&E) staining described briefly as follows. For IF-stained slides, standard indirect IF staining protocols were used, where unconjugated J533 antibody and AF488 donkey antimouse antibody were used for detection and imaging. H&E staining was performed on serial sections by the OHSU Histology Shared Resource. Both IF- and H&E-stained slides were imaged using the Zeiss AxioScan.Z1 microscope (Zeiss) at 10× magnification. Images of the entire field were stitched together using the ZEN software.

### 2.12 Statistical Analysis

All statistical analysis was performed using custom written MATLAB code. Tumor-to-normal adipose, muscle, and prostate tissue diagnostic detection ability was determined by ROC curves generated for the untargeted probe images, targeted probe images, and calculated DDSI images. The perfcurve function in MATLAB was used to calculate area under the curve (AUC) values on a pixel-by-pixel basis with individual pixel values for each tissue type used as the response variable input. A threshold variable was generated with a linearly increasing value from the minimum to maximum pixel intensity with 100 times fewer number of values than the total number of pixel values. The true positive rate (percentage of tumor pixels greater than threshold), false positive rate (percentage of normal pixels greater than threshold), ROC curves, and corresponding AUC values were then generated and plotted at each threshold value. Histogram plots for tumor-to-normal adipose, muscle, and prostate tissue were generated, on which the optimal threshold point determined via ROC curve analysis was plotted.

## 3 Results

### 3.1 In Vitro PSMA Expression of Prostate Cancer Cell Lines

**Fig. 1:**
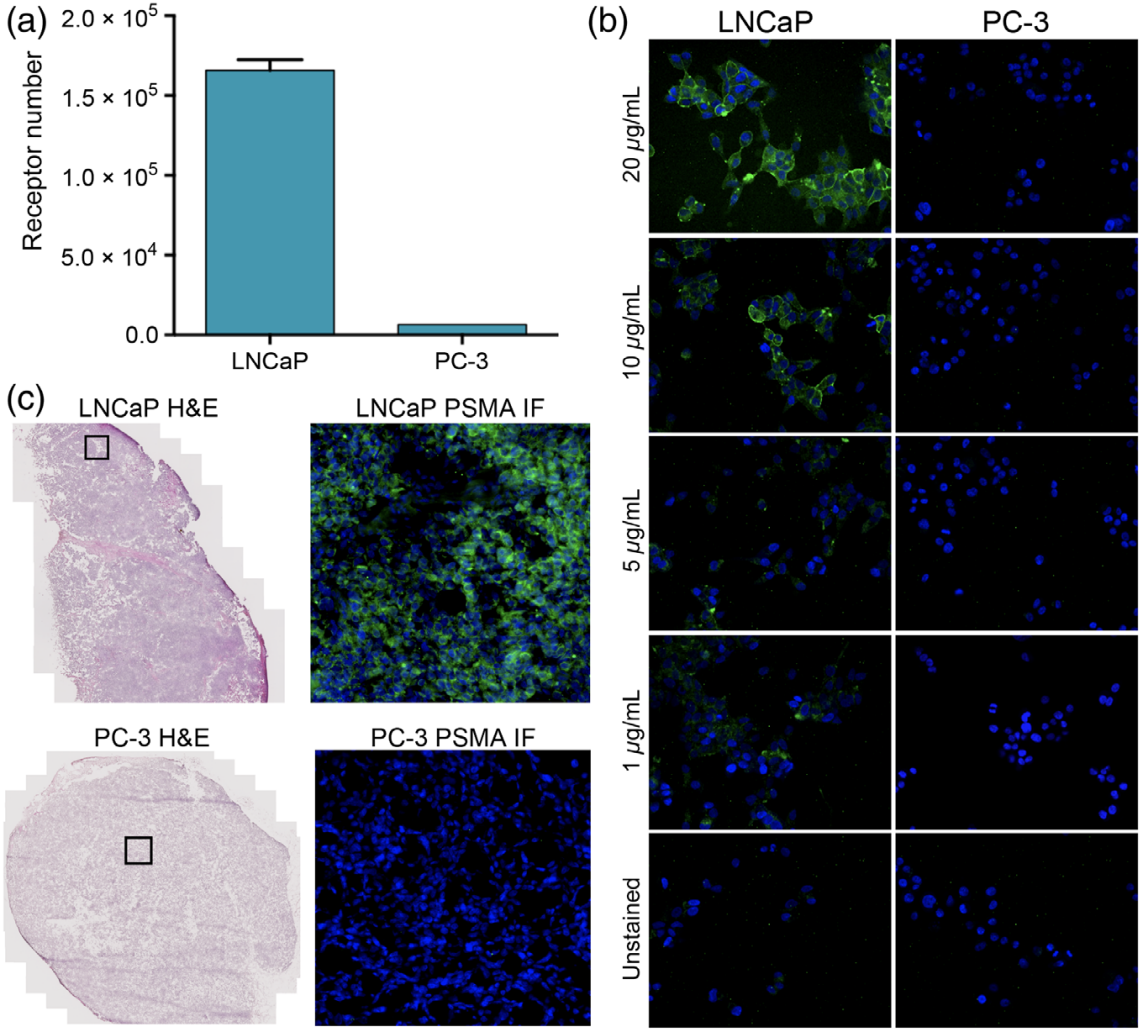
Prostate cancer cell line PSMA receptor quantification. (a) Flow cytometry-based quantification of LNCaP and PC-3 PSMA receptor number, where n=3 samples per cell line were used to quantify expression. (b) Fixed cell *in vitro* IF staining of PSMA expression was completed in LNCaP and PC-3 cells stained with J533-AF647 at varied concentrations (1, 5, 10, and 20  μg/mL). PSMA = green, Hoechst = blue. All images are shown with equivalent contrast and brightness. (c) Validation of PSMA expression in representative tumor tissue samples from LNCaP (top) and PC-3 (bottom) xenografts. Serial sections of representative resected specimens from each xenograft type were stained with gold standard H&E and indirect IF using the J533 antibody to validate PSMA expression in the model tissue. The black square on the H&E image represents the magnified region shown in the PSMA IF stained images. PSMA = green, DAPI = blue, H&E = hematoxylin and eosin.

Prostate cancer cell lines with varied PSMA expression *in vitro* were used, where the highly PSMA expressing LNCaP cell line ($1.7\times 10^{5}$ receptors per cell) was quantitatively compared with the minimally PSMA expressing PC-3 cell line [$6.6\times 10^{3}$ receptors per cell, Fig. 1(a)]. Adherent LNCaP and PC-3 cells were stained for PSMA *in vitro*, where the expected positive membrane-staining pattern for LNCaP cells was seen with decreasing intensity as primary antibody concentration decreased. PC-3 cells showed minimal staining even at the highest primary antibody concentration [Fig. 1(b)], demonstrating the PSMA antibody specificity. IF staining of the resected LNCaP and PC-3 xenograft tumors confirmed that PSMA expression was retained when the prostate cancer cell lines were grown as tumor xenografts [Fig. 1(c)].

### 3.2 PSMA DDSI Staining of Prostate Tumor Xenografts

**Fig. 2:**
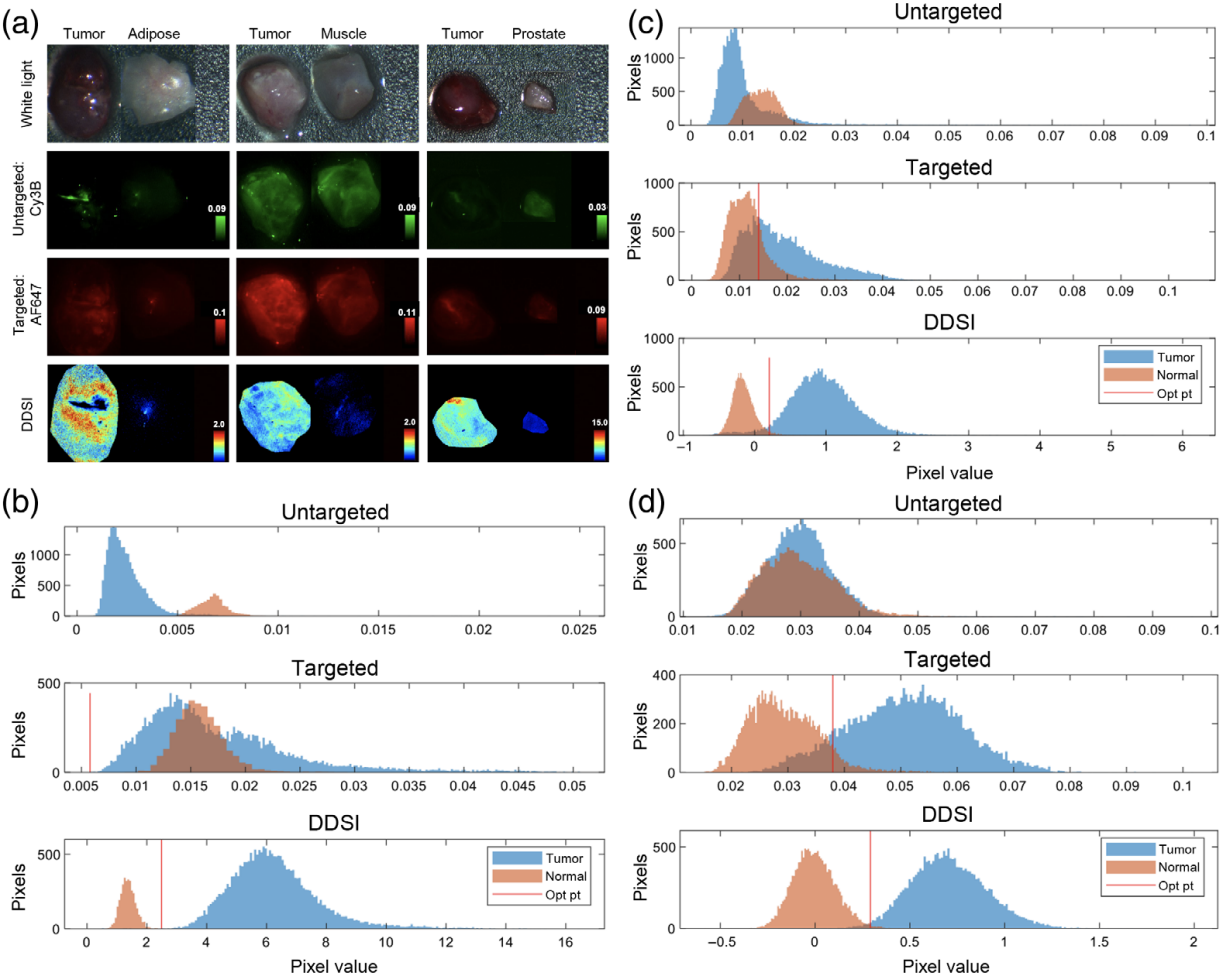
PSMA DDSI staining pattern and quantification in LNCaP tumor xenografts. (a) Representative white light, untargeted fluorescence (Cy3B), PSMA targeted fluorescence (AF647), and calculated DDSI images of LNCaP tumor versus normal adipose, muscle, and prostate tissues. LNCaP tumor and normal (b) prostate, (c) adipose, and (d) muscle tissue pixel intensity histograms for untargeted, targeted, and DDSI images following staining. All images and histograms are shown for a representative tumor and normal (i.e., prostate, adipose, and muscle) set of tissues and are representative of the data collected for n=6 tumor versus benign normal tissue pairs.

**Fig. 3:**
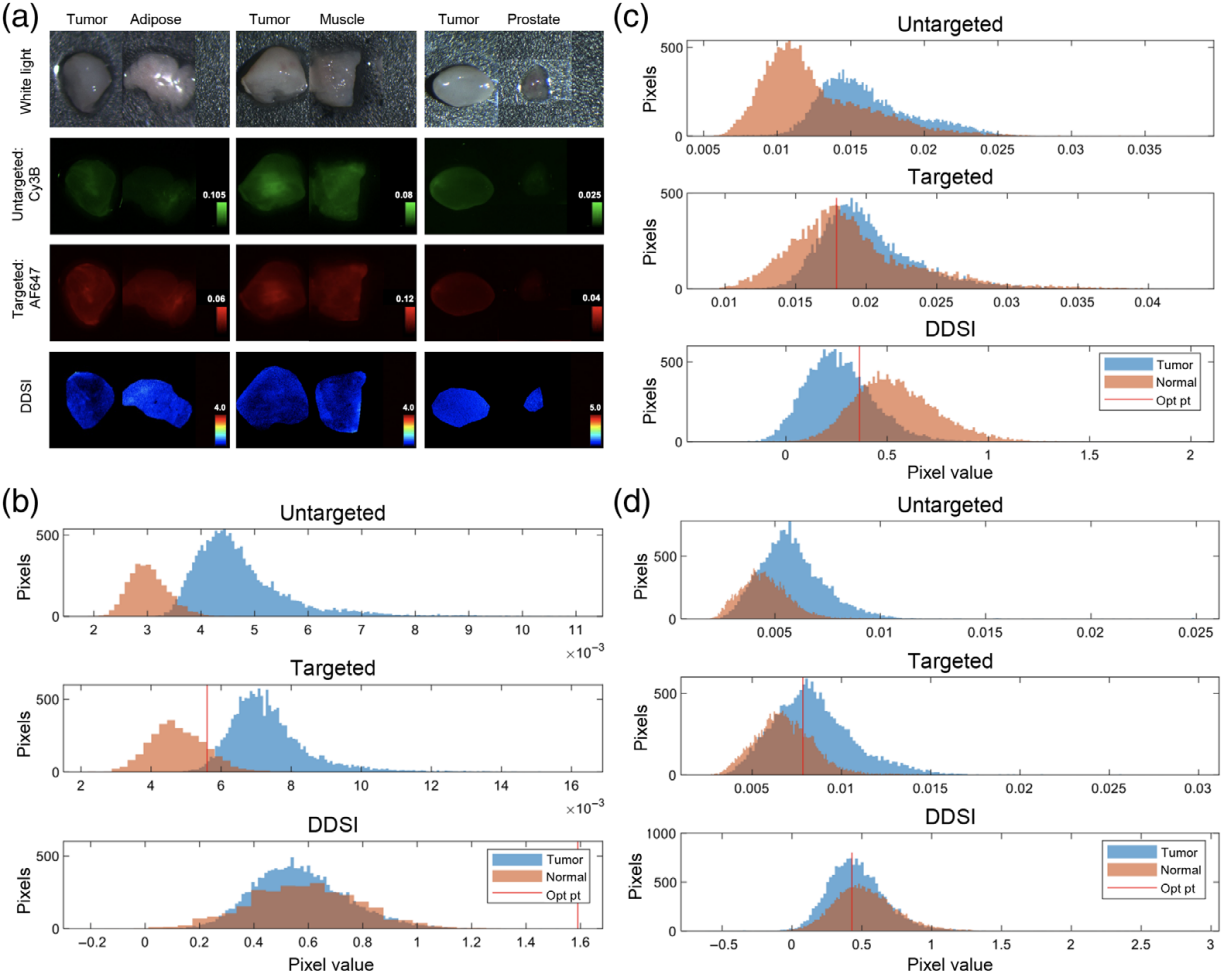
PSMA DDSI staining pattern and quantification in PC-3 tumor xenografts. (a) Representative white light, untargeted fluorescence (Cy3B), PSMA targeted fluorescence (AF647), and calculated DDSI images of PC-3 tumor versus normal adipose, muscle, and prostate tissues. PC-3 tumor and normal (b) prostate, (c) adipose, and (d) muscle tissue pixel intensity histograms for untargeted, targeted, and DDSI images following staining. All images and histograms are shown for a representative tumor and normal (i.e., prostate, adipose, and muscle) set of tissues and are representative of the data collected for n=6 tumor versus benign normal tissue pairs.

The PSMA positive prostate cancer-specific DDSI probe pair (J533-AF647 + DkRb-Cy3b) and the previously published DDSI staining protocol optimized for breast cancer were used to establish the accuracy of DDSI for prostate cancer detection. LNCaP (n=6), and PC-3 (n=6) tumor xenograft samples were stained with the DDSI staining protocol with corresponding normal adipose, muscle, and prostate tissues. Notably, little difference was seen between the targeted and untargeted probe uptake of the highly PSMA expressing LNCaP tumors versus the minimally PSMA expressing PC-3 tumors due to nonspecific probe uptake after topical administration. In addition, substantial untargeted probe uptake was seen in both tumor types as well as in the normal tissues [i.e., adipose, muscle, and prostate, Figs. 2(a) and 3(a)], again due to nonspecific probe uptake after topical administration. The similar nonspecific uptake between the targeted probes in the xenografts with substantial difference in PSMA expression as well as the substantial uptake of untargeted probe clearly demonstrated the need for ratiometric imaging for quantitative, molecular-specific margin assessment.

DDSI showed substantial contrast difference between the PSMA positive LNCaP tumors and their respective normal tissues [Fig. 2(a)]. In addition, DDSI showed substantial contrast difference between the highly PSMA expressing LNCaP and minimally PSMA expressing PC-3 tumors [Figs. 2(a) and 3(a)] due to the ratiometric correction for the nonspecific uptake patterns in resected tissues. Notably, LNCaP tumors presented with two phenotypes, which appeared as highly vascular versus relatively avascular, where the vascular LNCaP tumors showed lower uptake of the targeted and untargeted antibody stains compared to relatively avascular LNCaP tumors. However, these apparent fluorescence differences were largely corrected using the ratiometric DDSI protocol [Fig. 2(a)]. Notably, since visible fluorophores were utilized for the DDSI staining protocol, evaluation of PSMA expression was largely limited to the tissue surface, which is representative of the surgical margin to be assessed. Histograms of targeted pixel intensities across the sample set (LNCaP, n=6) showed strong overlap between the tumor and normal tissues [i.e., prostate and adipose tissues, Figs. 2(b) and 2(c)]. Of note, some targeted pixel intensity separation was seen between the LNCaP tumors and the normal muscle tissue [Fig. 2(d)]. However, when the DDSI pixel intensities were evaluated across the LNCaP sample set, substantial robust quantitative separation between tumor tissues and all normal tissue types (i.e., prostate, adipose, and muscle) was seen [Figs. 2(b)–2(d)].

The PC-3 tumor phenotypes were more homogenous, where similar targeted and untargeted uptake was seen between all representative tumors and stained normal tissues, with the exception of the prostate which had relatively low targeted and untargeted uptake. DDSI images showed minimal contrast difference between the PC-3 tumors and the normal tissues, since all tissues had relatively low PSMA expression [Fig. 3(a)]. Notably, histogram pixel intensities across the sample set (PC-3, n=6) showed separation of tumor to prostate signal for both the untargeted and targeted probe uptake [Fig. 3(b)]. By comparison, the histogram pixel intensities showed substantial overlap between the tumor and normal tissues (i.e., adipose and muscle) for both the targeted and untargeted uptake [Figs. 3(c) and 3(d)]. However, the DDSI histogram pixel intensities normalized all differences in signal between the PC-3 tumors and all normal tissue types (i.e., prostate, adipose, and muscle), the expected result given the low PSMA expression in these tissues [Figs. 3(b)–3(d)].

### 3.3 ROC Curve Analysis of DDSI Accuracy

**Fig. 4:**
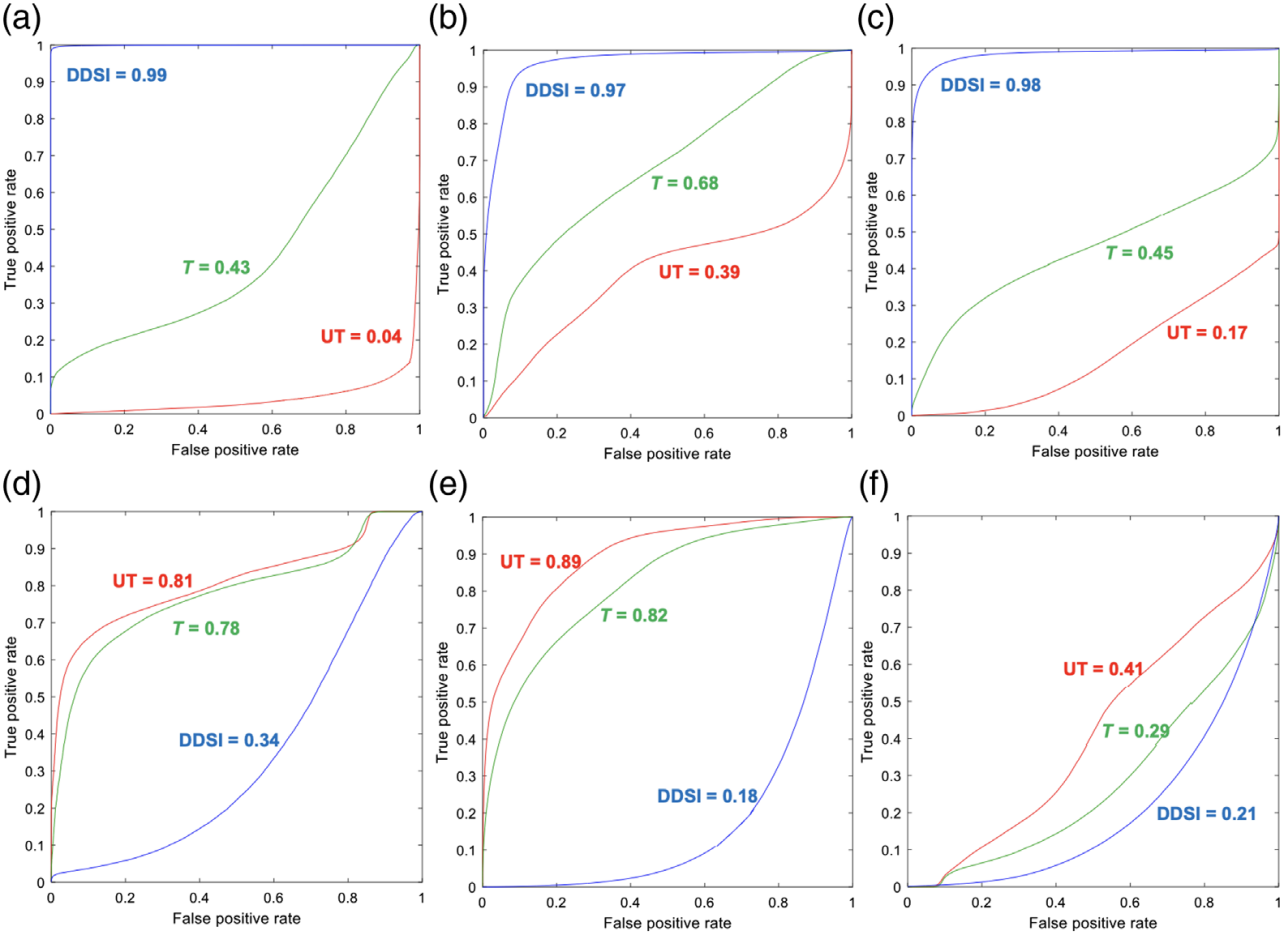
ROC curve analysis. ROC curves and calculated AUC values for untargeted, targeted, and DDSI images of LNCaP versus normal (a) prostate, (b) adipose, and (c) muscle following PSMA staining. ROC curves and calculated AUC values for untargeted, targeted, and DDSI images of the PC-3 tumors versus normal (d) prostate, (e) adipose, and (f) muscle following PSMA staining. All ROC curves and AUC calculations represent the collective data for all n=6 tumor versus normal tissue pairs. UT, untargeted; T, targeted.

The pixel intensity data from combined LNCaP tumors (n=6) and the combined PC-3 tumors (n=6) as well as their normal tissue counterparts were used to construct ROC curves of the targeted, untargeted, and DDSI contrast (Fig. 4). Minimal difference between the LNCaP tumors and their corresponding normal tissues was seen with either the targeted or untargeted probes alone. However, high diagnostic accuracy was obtained using the DDSI metric for LNCaP tumor to prostate (ROC AUC = 0.99), adipose (ROC AUC = 0.97), and muscle [ROC AUC = 0.98, Figs. 4(a)–4(c)]. Similarly, minimal difference between the PC-3 tumors and their corresponding normal tissue was seen with either the targeted or untargeted probes. However, given the minimal expression of the PSMA biomarker, using the DDSI metric for the PC-3 tumors did not improve the tumor to prostate (ROC AUC = 0.34), adipose (ROC AUC = 0.18) or muscle (ROC AUC = 0.21), which was not diagnostically useful [Figs. 4(d)–4(e)].

## 4 Discussion

The primary goals of this study were to extend the DDSI tumor margin assessment protocol to cancer types outside of breast cancer, where development and optimization were previously completed. Herein, the diagnostic utility of DDSI was specifically evaluated for use in prostate cancer using PSMA as the stained biomarker. PSMA was selected as the prostate cancer-specific biomarker since >90% of all primary prostate cancer lesions, tumor positive lymph nodes, and metastases overexpress this transmembrane protein.^47^^–^^51^ Prostatectomy—the current primary definitive therapy for localized prostate cancer—with positive cancer margins increases morbidity and mortality for patients by requiring further adjuvant treatments including combination therapy of radiation, ADT, chemotherapy, and/or bone-directed therapy.^3^ Fluorescence guided resection techniques have been shown to improve margin assessment using molecularly specific probes; however, clinical translation of contrast agents for *in vivo* administration is still challenging given the long and costly regulatory pathway. To circumvent these challenges, a novel DDSI staining protocol was developed and optimized that could be utilized on resected specimens from multiple cancer types without requiring either the contrast agents or FGS system to make contact with the patient, thus paving the way for rapid clinical translation of molecularly specific margin assessment.

A previously optimized DDSI protocol showed differentiation between malignant breast tissue and normal benign tissues with high sensitivity and specificity.^36^^,^^37^^,^^42^^,^^44^ In this paper, the DDSI protocol was extended to prostate cancer models to evaluate its utility to differentiate malignant prostate tissues from clinically relevant normal tissue (i.e., prostate, adipose, and muscle). The diagnostic potential of the DDSI technique was assessed using ROC curve analysis and AUC values to quantitatively compare between normal tissues, PSMA(+) tumors, and PSMA(−) tumors. PSMA expression levels between model prostate cancer cell lines were quantified [Fig 1(a)] and visualized *in vitro* [Fig 1(b)] and *ex vivo* [Fig. 1(c)] to verify that biomarker levels were consistent following tumor xenograft growth in mouse models.

The optimized DDSI staining protocol was readily extended to prostate cancer using PSMA as the staining biomarker. DDSI showed improved tumor to normal tissue (i.e., prostate versus adipose or muscle) differentiation as compared to using the targeted probe (i.e., PSMA probe) alone in highly PSMA expressing LNCaP tumor xenografts (Fig. 2). ROC AUC was improved from 0.43 to 0.68 using the targeted probe alone to 0.97 to 0.99 using the DDSI method [Figs. 4(a)–4(c)], demonstrating the quantitative improvement the ratiometric DDSI approach enabled over use of the targeted agent alone. The molecular specificity for the DDSI stained biomarker was further demonstrated using minimally PSMA expressing PC-3 tumors. These tumors had low biomarker expression and showed no appreciable signal difference via DDSI (Fig. 3). Quantification using ROC AUC showed the PSMA targeted probe alone had a wide AUC variance ranging from 0.29 to 0.81. However, these values were more similar using the DDSI approach ranging from 0.18 to 0.34 and showing lack of contrast due to lack of biomarker expression [Figs. 4(d)–4(f)]. Interestingly, LNCaP tumor xenografts grew with two distinct phenotypes which appeared as highly vascular (i.e., dark red in color, Fig. 2(a), white light, left and right panels) and relatively avascular (i.e., white to pink in color, Fig. 2(a), white light, middle panel), where the vascular LNCaP tumors appeared to have lower uptake of the targeted and untargeted probes compared with avascular LNCaP tumors [Fig. 2(a), left and right panels of targeted and untargeted images show lower tumor uptake than middle panel]. However, this observed fluorescence difference could be attributed to differences in tissue optical properties rather than actual probe uptake given the difference in blood content between tumor phenotypes, especially since visible fluorophores were used to label the targeted and untargeted probes, which have spectral overlap with the absorption of hemoglobin. Notably, the fluorescence difference seen between the highly vascular and relatively avascular tumor phenotypes was largely corrected using the ratiometric DDSI protocol, showing the potential utility of DDSI for biomarker-specific quantification in tissues with varied optical properties due to differing hemoglobin concentrations.

The only intraoperative margin assessment methodology developed and in use for prostatectomy at select hospitals is FSA, which adds 20 min to the surgical time in 90% of cases on average^58^ and required a trained pathologist in the OR. The use of the optimized DDSI protocol, which requires a total of 8 min,^37^ would alleviate some of the current challenges faced during intraoperative tumor margin assessment using FSA. Specifically, it could eliminate the need for an OR pathologist as the surgical team could use the DDSI fluorescence to quantify tissue biomarker expression as a surrogate for positive margins, providing the ability to resect additional tissues at the time of surgery and potentially prevent the need for repeat surgeries. All tissues would be evaluated using DDSI in the OR without destruction and thus would not affect the clinical workflow, where the gold standard for margin assessment would still be clinical histopathology. Preservation of the resected specimen would allow for postoperative gold standard histopathology that requires at least 24 h to result and can also be readily co-registered to the corresponding DDSI maps. Thus, intraoperative rapid margin assessment could enable surgeons to make decisions about removal of additional tissues to improve margin negative surgical procedures in <10  min from tissue removal from the body. This could improve patient cancer control and therapeutic outcomes by decreasing the rate of reoperation or further adjuvant therapies. The DDSI methodology also provides advantageous high-resolution, wide-field visualization of the entire tissue specimen via the generation of accurate tumor biomarker expression maps, which is not available to conventional margin detection technologies.

Limitations to the current work include the separation of tissue types into discrete entities (i.e., tumor, adipose, muscle, and prostate), which would be a heterogeneous mixture in clinical samples. In addition, a limited number of prostate cancers do not overexpress PSMA and thus additional biomarker development would be required to facilitate diagnostic DDSI for margin status assessment in all prostatectomy cases. In future studies, clinical sample staining will be evaluated to assess the diagnostic accuracy of the DDSI methodology in mixed, heterogeneous samples, and additional biomarkers will be evaluated for low PSMA expressing prostate cancers. In summary, the DDSI methodology could be extended to evaluate margin status across additional cancer types, which would enhance the diagnostic and surgical capability of DDSI, improving patient outcomes.

## Data, Materials and Code Availability

All relevant data, materials and software code used in this study are available upon reasonable request from the corresponding author.
